# In vitro anti-*Onchocerca ochengi* activities of extracts and chromatographic fractions of *Craterispermum laurinum* and *Morinda lucida*

**DOI:** 10.1186/1472-6882-14-325

**Published:** 2014-09-01

**Authors:** Moses Samje, Jonathan Metuge, James Mbah, Brice Nguesson, Fidelis Cho-Ngwa

**Affiliations:** Department of Biomedical Sciences, Faculty of Health Sciences, University of Bamenda, PO Box 39, Bambili, Cameroon; Department of Biochemistry and Molecular Biology, Faculty of Science, University of Buea, P.O. Box 63, Buea, Cameroon; Department of Chemistry, Faculty of Science, University of Buea, P.O. Box 63, Buea, Cameroon

**Keywords:** Onchocerciasis, Medicinal plants, Toxicity, Phytochemical analysis

## Abstract

**Background:**

Onchocerciasis caused by *Onchocerca volvulus* is the world’s second leading infectious cause of blindness. There is currently no cure for the disease. Ivermectin, the current drug of choice is only microfilaricidal and suboptimal response to it is increasingly being reported. Thus, in contributing to the search for a cure, crude extracts and chromatographic fractions of *Craterispermum laurinum* and *Morinda lucida* were screened in vitro, against the bovine and most popular model of the parasite, *Onchocerca ochengi*.

**Methods:**

Extracted parasites were cultured in RPMI-1640 based media for 05 days in the presence of control drugs, test drugs or drug diluents only. Microfilarial motility was scored using microscopy while adult worm viability was determined biochemically by MTT/formazan colorimetry. Cytotoxicity and acute toxicity of active fractions were tested on monkey kidney epithelial cells (LLCMK2) and in Balb/c mice, respectively.

**Results:**

Out of the 18 extracts screened, the methanolic extracts of the leaves of both plants recorded the highest activities against both the microfilariae (IC_100_ of 125 μg/ml for both extracts) and adult worms (IC_100_ of 250 μg/ml and 500 μg/ml for *M. lucida* and *C. laurinum* respectively). The most active chromatographic fraction was obtained from *M. lucida* and had an IC_50_ of 7.8 μg/ml and 15.63 μg/ml on microfilariae and adult worms respectively, while the most active fraction from *C. laurinum* had an IC_50_ of 15.63 μg/ml and 46.8 μg/ml, respectively on microfilariae and adult worms. The 50% cytotoxic concentration (CC_50s_) on LLCMK2 cells ranged from 15.625 μg/ml to 125 μg/ml for the active fractions. No acute toxicity was recorded for the extracts from both plants. Phytochemical analysis of the most active fractions revealed the presence of sterols, alkaloids, triterpenes, saponins and flavonoids.

**Conclusions:**

This study validates the use of these plants by traditional health practitioners in managing the disease, and also suggests a new source for isolation of potential lead compounds against *Onchocerca volvulus*.

## Background

Human onchocerciasis (river blindness) caused by the filarial worm, *Onchocerca volvulus* is one of the neglected tropical diseases of major public health concerns [1]. The disease is the world’s second leading infectious cause of blindness with over 37 million patients and a risk population of over 120 million [2]. Hyper endemic villages can have infection rates of close to 100%, where up to 10% of an entire village may be blind due to the disease. Close to 99% of all patients live in Tropical Africa [3, 4]. Pathologically, the disease is associated with extensive and disfiguring skin changes, musculoskeletal complaints, weight loss, and changes in the immune system [5]. In addition to its severe pathological effect, it causes grave socio-economic problems and life-long human suffering [6].

Two major strategies employed in the control of onchocerciasis are mass treatment of infected people with ivermectin and the elimination of the *Simulium* vector [7, 8]. Despite the successes registered in reducing the disease burden, total elimination has not been achieved due to pitfalls in the control programmes. At present, only ivermectin (Mectizan®, Merck) is recommended for chemotherapy and for mass drug administration. Although this drug has been shown to significantly reduce transmission of the disease, its filaricidal effect is limited only on the juvenile form of the parasite [9, 10]. Studies have revealed that treatment of some *Onchocerca* patients with ivermectin who are co-infected with *Loa loa* may result in adverse effects, which ranged from fatigue to consciousness disorders and death [11, 12]. Therefore, the ideal drug for onchocerciasis would be inactive against the microfilariae of *Loa loa*. Suboptimal response to ivermectin has also been reported in different studies [13–16]. Thus, the search for new, safe and efficacious onchocerciasis drugs is imperative.

Herbal medications play a central role in the cure of several diseases particularly in developing countries [17, 18]. In some Asian and African countries, 80% of the population depends on traditional medicine for primary health care, and herbal medicines are the most lucrative form of traditional medicine, generating billions of dollars in revenue [19]. The anti-filarial activities of some plant extracts have been documented [20–23]. *M. lucida* and *C. laurinum* (family: *Rubiaceae*) have been shown to express anti-bacterial activity on a range of bacteria [24, 25]. Other separate studies have revealed that *M. lucida* possess anti-plasmodial activity [26], suppressed *Trypanosoma brucei* parasites [27] and have hypoglycaemic activity [28] *. Onchocerca ochengi,* found exclusively in cow is the closest relative to the medically important *O. volvulus*. Similarities lie in them sharing the same vector, their microfilariae being equally sensitive to ivermectin and their adult worms found in the sub-cutaneous/intradermal nodules [29]. Since the bovine parasite species is relatively more abundant in Africa and also cheap to obtain, it is thus a model of choice in anti-onchocercal drug screens. In an attempt to contribute to anti-onchocercal lead development, we herein report on in vitro filaricidal properties of both crude extracts and chromatographic fractions of extracts of *C. laurinum* and *M. lucida* against *Onchocerca ochengi* parasite stages. Additionally, we report on the cyto- and acute toxicity profiles of the best extracts and fractions of the two plants, thereby initiating a novel lead compound discovery endeavour.

## Methods

### Collection and identification of plant materials

Various plant parts (leaves, barks and roots) of *C. laurinum* were collected from Finge village of the Bambui Health District in the North West Region of Cameroon in June 2010, while *M. lucida* plant parts were collected from Buea at the foot of Mount Fako, Cameroon in January 2011. The plants were selected based on ethnopharmacological information about them. The plants were identified and authenticated by Mr. Paul Mezili of the National Herbarium, Yaounde, Cameroon and given the voucher number (Poir) Benth No 3781/SRFK for *C. laurinum* and Benth No 2615/SRFK for *M. lucida*. Locally, *C. laurinum* is called “sarkaatari”.

### Preparation of crude extracts and chromatographic fractions

All the plant parts collected were air dried, then ground to fine powder. The ground materials were weighed and sequentially submerged and macerated for a total of 72 hours in three solvents thus: hexane (HEX), methylene chloride (MC), and methanol (MeOH). For each solvent, the maceration was repeated twice. The mixture was filtered and the filtrate concentrated using a rotary evaporator (BUCHI Rotavapor R-200, Switzerland) at appropriate temperatures. The concentrate were recovered with methylene chloride and allowed to stand open at room temperature until all the residual solvents had evaporated. The dried crude extracts were stored at -20°C until needed for the assays.

Bioassay-guided fractionation was done on the most active crude extracts. Each of the latter extract was fixed on celite and fractionated using vacuum liquid chromatography on silica gel and then eluted with a continuous gradient of ethyl acetate (EtOAc [0–80%]) in hexane, followed with a gradient of methanol (MeOH [0-40%]) in methylene chloride. Collected fractions were pooled on the basis of their thin layer chromatographic (TLC) profiles.

### Isolation and culture of *O. ochengi*adult worms (macrofilariae)

This was done by the method of Cho-Ngwa et al. [22] with slight modifications. Briefly, fresh pieces of umbilical cattle skin containing large amount of palpable nodules were brought from local slaughterhouses to the laboratory. The skin was thoroughly washed successively with tap and distilled water, drained and totally covered with 70% ethanol which was allowed to evaporate on its own in a laminar flow hood sterile environment. Pale orange-yellow *O. ochengi* adult worm masses containing essentially viable adult female and male worms were recovered by careful dissection of the nodules using sterile razor blade. The extracted worms were immediately submerged into complete culture medium (CCM) (RPMI-1640 supplemented with 25 mM HEPES, 2 g/L sodium bicarbonate, 20 mM L-glutamine, 10% new born calf serum [SIGMA, USA], 200 units/ml penicillin, 200 μg/ml streptomycin and 2.5 μg/ml amphotericin B [Sigma, USA], pH 7.4) in 24 wells. The viability of worms was determined by microscopic examination using an inverted microscope (euromex, Holland). The cultures were incubated at 37°C under an atmosphere of 5% CO_2_ in humidified air in a HERACELL-150 CO_2_ incubator (Thermo Electron, Germany) for 05 days post addition of drug.

### Extraction of microfilariae

Fresh and cleaned infected cattle skin was sterilized as above and tautly attached onto a sterile wooden board. A portion of the skin was then carefully shaved with razor blade, rinsed with distilled water and completely covered with 70% ethanol. The ethanol was allowed to evaporate freely and completely in a laminar flow hood. The skin was then firmly attached onto an autoclaved cylindrical piece of wood and close crisscross cuts of about 0.5-1 mm apart were made into the epidermal and dermal layers, over the entire skin surface. The entire assembly was transferred into a sterilized glass cylinder and an appropriate volume of CCM was added to just cover the entire skin. The assembly was incubated for 4 – 6 hours at room temperature. The emergent and highly motile microfilariae (mfs) were concentrated by centrifugation (400 × *g* for 10 minutes) and quantified microscopically. Then, 100 μl of CCM containing 15–20 mfs was transferred into each well of a 96 well microtitre culture plates. After addition of drugs, the mfs were cultured at 37°C under an atmosphere of 5% CO_2_ in humidified air in a HERACELL-150 CO_2_ incubator (Thermo Electron, Germany).

### Preparation of mammalian cells for microfilarial cultures and cytotoxicity assay

Monkey kidney epithelial cells (LLCMK2) purchased from the American Type Culture Collection (ATCC, Virginia, USA) was proliferated in complete culture media at 37°C and 5% CO_2_. Once the cells became fully confluent, the old media was decanted and the cells were dislodged with 0.125% trypsin and 0.5 mM EDTA in serum-free RPMI-1640 culture medium. The dislodged cells were re-suspended in 10 mL of complete culture medium and centrifuged at 560 × g for 10 minutes to get rid of the trypsin. The last procedure was repeated once. The cell suspension (100 μL/well) was transferred into 96-well microtitre culture plates and kept in the CO_2_ incubator for cells to grow and become fully confluent, usually taking 3–5 days depending on the initial concentration of cells. LLCMK2 cells served as feeder layer for mfs cultures and also for cytotoxicity studies. The selectivity index (SI) of each extract was calculated as the ratio of CC_50_ of drug to these mammalian cells to the IC_50_ of the drug on the parasites.

### Preparation and in vitro screening of crude extracts and fractions

A stock solution of 25 mg/ml crude extract in ≥99.9% sterile dimethyl sulfoxide (SIGMA, USA) was prepared. Adult worm assays were conducted in 24-well plates (2 mL/well) (NUNC, USA) for 5 days without any change of medium. On the other hand, all mfs-only assays were conducted in 96-well microtitre plates (200 μL /well) that contained confluent feeder layer of the monkey kidney cells. The media used in preparing the feeder cell layer was removed by swift decantation before fresh medium and worms for drug testing were added. Assays were run for 5 days post-addition of extract without any change of medium.

### Primary screen for adult worm and microfilariae

This was done in order to eliminate inactive extracts. Each extract was dissolved to 2X its final test concentration in CCM in a single sterile well before distribution into replicate wells. In this regard, 1 ml per well or 100 μl per well for adult worm or mfs respectively, were transferred into replicate culture wells, each already containing either adult worm in 1 ml of CCM or mfs in 100 μl of CCM, to give a final volume of either 2 ml for the adult worm assay or 200 μl for the mfs assay. The crude extracts were tested in quadruplicate wells (for adult worms) and in duplicate (for mfs) at a single concentration of 500 μg/mL. A compound (BTU55261) known to be active (unpublished) was used as the positive control for the adult worm assay, while ivermectin (IVM) was used as a positive control for the mfs assay. BTU55261 produces 100% inhibition of formazan formation in adult worms on day 5 at a concentration of 2.5 μg/ml, while IVM totally inhibits mfs motility at 10 μg/ml by day 5. Negative controls were treated in the same way as the test samples, received the drug diluents (≤2% DMSO in RPMI), but with no drug added.

Male worm motility inhibition was scored using an inverted microscope and was recorded as 100% (complete inhibition), 75% (only head or tail of worm moving or vibrating), 50% (worm sluggish) or 25% (little change in motility), 0% (no observable inhibition of motility). Biochemical evaluation of adult worm viability was done using the MTT/formazan colorimetric assay [30, 31]. The worms were placed under sterile conditions in a well of a 48-well plate containing 500 μL/well of 0.5 mg/mL MTT (Sigma, USA) in serum free media and then incubated under the culture conditions for 30 minutes. Thereafter, the worms were blotted individually on tissue paper and colour development of the test compared with the controls. MTT, a pale yellow compound is reduced to a dark blue product, formazan by the living cells of the worms. Adult worm viability was taken as mean percent inhibition of formazan formation relative to negative control at 120 hours post addition of drug. Inhibition of formazan formation by extracts relative to the negative controls showed that an extract was either active (90% or greater mean inhibition of formazan formation), moderately active (50 - 89% mean inhibition of formazan formation) or inactive (<50% mean inhibition of formazan formation). Viability of mfs was assessed by reading their mean motility scores every 24 hours (post addition of drug) for 120 hours on the same scale as for adult male worms. An extract was considered active on the mf if there was a 100% reduction in mfs motility; moderately active if there was a 50-99% mean reduction in mfs motility and inactive if there was less than 50% mean inhibition of motility compared to untreated controls of the same experiment.

### Secondary screen

This was done in order to confirm the activity of extracts that were promising in the primary screen, as well as to determine the IC_50_ and selectivity index (SI) values. All the active extracts that showed 100% macrofilaricidal and/or microfilaricidal properties were re-tested as in the primary screens and at serial dilutions of 8 different concentrations starting from 500 μg/mL down to 3.91 μg/mL. The graphical analysis was done using Microsoft Excel 2007 (Microsoft Corporation, USA).

### Acute toxicity of active extracts in mice

The test was conducted following the European Community Guidelines for protection of animals used for experimental purposes [32] and respecting the 3Rs (Replacement, Refinement and Reduction) of Animals in Research [33]. Currently, there is no law governing the use of Balb/C mice for research purposes in Cameroon. Mice used in the study were commercially gotten from Centre Pasteur, Yaoundé. Nulliparous, non-pregnant 10 weeks old female Balb/c mice were weighing 22.4 ± 1.5 g were randomly selected and kept in their cages for 5 days before dosing [34]. The test was done for the methanolic extracts of *C. laurinum* and *M. lucida* as well as for fractions that demonstrated an IC_50_ of ≤ 31.25 μg/ml on mfs. The equivalent of 5 times IC_50_ of each substance was injected intraperitoneally into separate groups of 6 mice while 6 control mice were injected with an equal volume of diluent (2% DMSO in normal saline). Briefly, considering the 5 × IC_50s_ of 93.75 μg/ml for the *C. laurinum* extract, 10.3 mg of the extract was dissolved in 100% DMSO and then diluted in normal saline such that the final DMSO concentration was 2%. The diluted extract was then injected into the chosen mice. The same procedure was repeated for all the selected active fractions. Following dosing, the animals were observed every 30 minutes for the first 4 hours. Thereafter, the physical activity, weight of food and the volume of water consumed, changes in skin and fur, tremors, convulsions, diarrhea, sleep, coma and death were noted daily for the 14 day study period.

### Phytochemical analysis

Following screening, phytochemical derivatives found in the most active fractions were investigated by standard methods [35]. Briefly, the presence of alkaloid was determined using the Drangedoff’s reagent while the presence of sterols and triterpenes was determined by the Liebermann Buchard reaction. For the presence of flavonoids, the extract was dissolved in methanol and 2–3 mL of concentrated HCl was added. A spatula full of magnesium turnings was then added and the mixture observed for effervescence. The presence of saponins was determined by dissolving a trace amount of the extract in water and shaking thoroughly. Frothing which persisted on warming was observed and taken as preliminary evidence. Test for Tanins was done by adding 10 ml of distilled water to 2 g of plant extract, stirred, filtered and ferric chloride later added to the filtrate.

## Results

### Activity of crude extracts of both plants on adult worm and microfilariae

A total of 18 crude extracts were prepared from the two plants (9 from each) using solvents of different polarities (hexane, methylene chloride and methanol). Results of the primary screen showed that the methanol extracts of the leaves of both plants had the highest anti-parasite activity. By contrast, the methanol extracts of barks, roots, as well as the hexane extracts and methylene chloride extracts of both plants were inactive. Consequently, further testing on these extracts was discontinued. Table 1 summarizes the effect of the methanolic extracts of both plants on adult worm and mfs.

**Table 1:** **Dose-dependent effect of crude extracts of** ***C*** **.** ***laurinum*** **and** ***M. lucida*** **on male and female adult worms and microfilariae**

Concentration of extract (μg/mL)	% inhibition of adult male worm motility		% inhibition of formazan formation by female worm		% inhibition of mfs motility	
	CL _LMeOH_	ML _LMeOH_	CL _LMeOH_	ML _LMeOH_	CL _LMeOH_	ML _LMeOH_
500	100	100	100	100	100	100
250	50	100	50	100	100	100
125	50	50	50	50	100	100
62.5	0	0	0	0	25	50
31.25	0	0	0	0	0	25
15.63	0	0	0	0	0	0
0	0	0	0	0	0	0
Positive control	100	100	100	100	100	100
Negative control	0	0	0	0	0	0

(CL_LMeOH_: methanolic extract of *C. laurinum* and ML_LMeOH_: methanolic extract of *M. lucida*).

**Figure 1:**
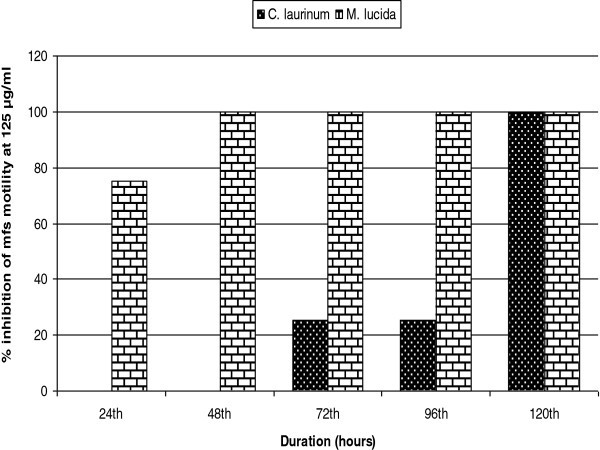
**Time-dependent inhibition of mfs motility at 125 μg/ml by the methanolic extract of** ***M. lucida*** **and** ***C. laurinum.***

The SIs of CL_LMeOH_ and ML_LMeOH_ on adult male and female worms was 1 while those of CL_LMeOH_ and ML_LMeOH_ on mf were respectively 2.6 and 2 and that of ivermectin in the same experiment was 1.0. This was not surprising since ivermectin kills *Onchocerca* mfs in vitro within 5 days only at a much higher concentration relative to the therapeutic dose in humans. Although there was 100% inhibition of mfs motility on day 5 for extracts of both plants at 125 μg/ml, *M. lucida* demonstrated more rapid activity in time-dependent studies (Figure 1). Complete inhibition of motility was observed with *M. lucida* by the 48th hour unlike *C. laurinum* which showed full activity only at the 120th hour.

### Activity of chromatographic fractions of *C. laurinum*on microfilariae and adult worms

**Figure 2:**
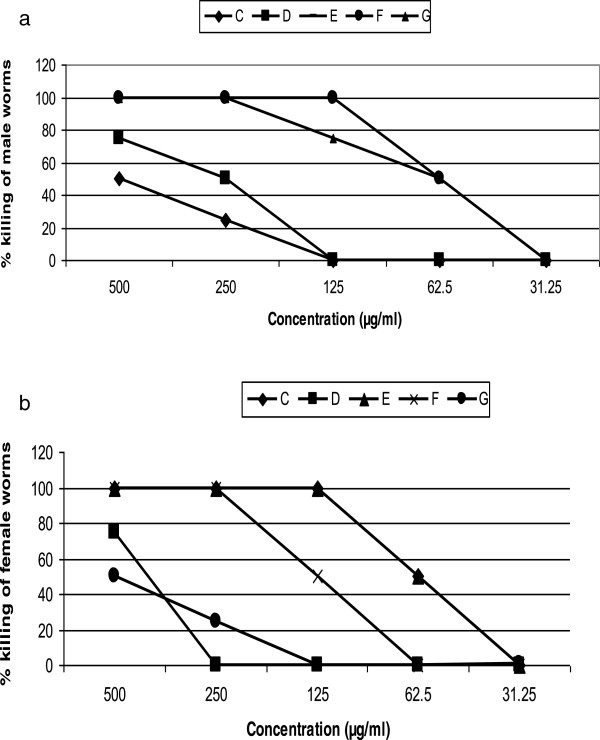
**Percent killing of male adult worms (a) and female adult worms (b) by active fractions from** ***C. laurinum.***

**Figure 3:**
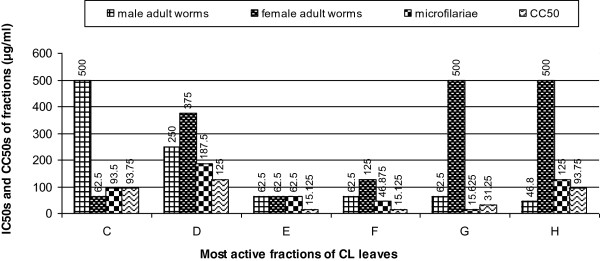
**IC**_**50s**_ **and CC**_**50s**_ **of most active fractions of** ***C. laurinum*** **on respectively mfs, adult worms and LLCMK2 cells.**

Nine fractions; A, B, C, D, E, F, G, H, I were sequentially obtained and tested on both the juvenile and adult form of the parasite. The first and last fractions (A and I) were inactive on both the mfs and adult worms. The highest activity on mfs (IC_100_ of 31.25 μg/ml and IC_50_ of 15.625 μg/ml) was observed with fraction G (Table 2). Except for fractions B and H, there was 100% inhibition of mfs motility by the 24th hour of culture in the presence of the active fractions at the highest concentration of 500 μg/ml. Four fractions (A, B, H and I) showed no activity on the adult female worms. Out of these, Fraction H demonstrated moderate activity on the adult male worms while the rest were inactive on the male worms. Fraction E inhibited motility and formazan formation in the male adult and female adult worms respectively at the same concentration (Figure 2 a and b). A comparison of the IC_50s_ of the adult worms and mfs and the CC_50s_ of the most active fractions is shown in Figure 3.

**Table 2:** **Chromatographic profile and activity of nine fractions of CL**_**LMeOH**_ **on microfilariae motility**

Eluent	Combined Fractions: code	% inhibition of microfilarial motility on day 5 concentration of extracts (μg/ml)								
Hex:EtOAc		500	250	125	62.5	31.25	15.63	7.8	0	IC _50_of mfs
100:0	1-14: A	0	0	0	0	0	0	0	0	/
90:10	15-20: B	100	50	0	0	0	0	0	0	250
80:20	21-28: C	100	100	100	0	0	0	0	0	93.5
70:30	29-39: D	100	100	25	25	0	0	0	0	187.5
60:40	40-60: E	100	100	75	50	25	0	0	0	62.5
40:60	61-67: F	100	100	100	100	75	50	0	0	15.63
CH_2_Cl_2_:MeOH										
20:80	68-78: G	100	100	100	100	100	50	0	0	15.63
95:5	79-99: H	100	75	50	25	0	0	0	0	125
0:100	100-108: I	0	0	0	0	0	0	0	0	/

Hex: hexane, EtOAc: ethyl acetate, TLC: thin layer chromatography, CH_2_Cl_2_: methylene chloride, MeOH: methanol.

### Activity of chromatographic fractions from the methanol extract of *M. lucida*on microfilariae and adult worms

**Figure 4:**
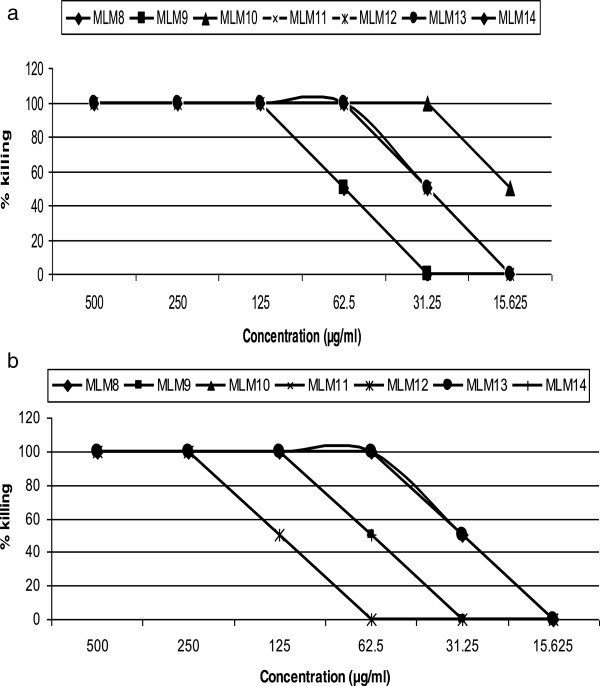
**Percent killing of male adult worms (a) and female adult worms (b) by active fractions from** ***M*** **.** ***lucida.***

**Figure 5:**
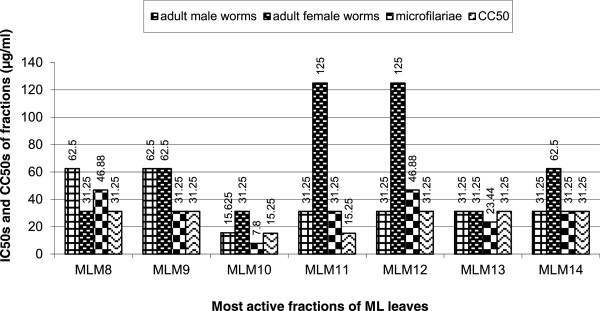
**IC**_**50s**_ **and CC**_**50s**_ **of active fractions of** ***M. lucida*** **on mfs, adult worms and LLCMK2 cells.**

**Figure 6:**
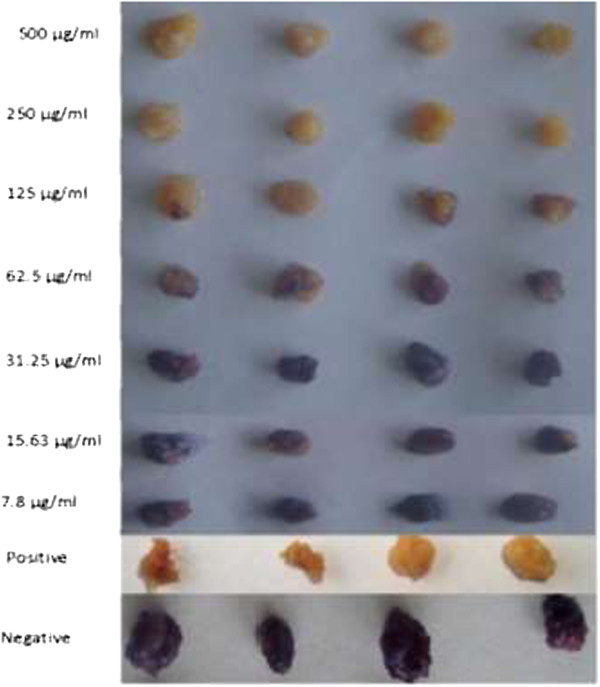
**Variation in the conversion of MTT salt to formazan by adult female worms.**

One hundred and fifty fractions of 500 mL each were collected using hexane:ethyl acetate and methylene chloride:methanol eluent. The fractions were pooled on the basis of their TLC profiles to give a total of seventeen combined fractions (MLM 1 – 17). Out of these 17, four of them showed moderate activity on mfs but were inactive on either the adult male or female worms while the rest exhibited full activity on mfs at a concentration of 500 μg/ml by day 5. The dose-dependent result of the active fractions on mfs by the 120th hour of culture is shown on Table 3. Fraction MLM10 was the most active with an IC_100_ of 15.63 μg/ml and an IC_50_ of 7.8 μg/ml. Additionally, the fractions also exhibited a time dependent inhibition of mfs and adult male motility. Out of the 17 fractions, seven showed activity on the adult worms (Figure 4a and b) while 5 were moderately active. Only two of the seven active extracts exhibited effect on both the male and female worms at the same concentration. The general trend observed for the seven most active fractions was that they were all almost more active on the male adult worms and mfs than their adult female counterpart (Figure 5). Figure 6 shows a colored picture on the varying degree of killing of adult worms by fraction MLM11. This dose-dependent disparity in the conversion of MTT to formazan is a reflection of adult worm viability.

**Table 3:** **Chromatographic profile and activity of seventeen fractions from ML**_**LMeOH**_ **on microfilariae motility**

Eluent	Combined Fractions:code	% inhibition of microfilariae motility on day 5 concentration of extracts (μg/ml)								
Hex:EtOAc		500	250	125	62.5	31.25	15.63	7.8	0	IC _50_of mfs
100:0	1-13: MLM1	100	50	0	0	0	0	0	0	250
95:5	14-16: MLM2	50	0	0	0	0	0	0	0	500
	17-24: MLM3	100	100	50	0	0	0	0	0	125
90:10	25-32: MLM4	50	0	0	0	0	0	0	0	500
80:20	33-40: MLM5	100	75	0	0	0	0	0	0	187.5
	41-49: MLM6	50	25	0	0	0	0	0	0	500
70:30	50-54: MLM7	100	75	50	0	0	0	0	0	125
	55-61: MLM8	100	100	100	50	25	0	0	0	62.5
60:40	62-65: MLM9	100	100	100	100	50	0	0	0	31.25
	66-74: MLM10	100	100	100	100	100	100	50	0	7.8
50:50	75-86: MLM11	100	100	100	100	50	50	25	0	15.63
40:60	87-100:MLM12	100	100	100	50	0	0	0	0	62.5
20:80	101-109:MLM13	100	100	100	100	100	25	0	0	23.44
	110-117:MLM14	100	100	100	100	50	0	0	0	31.25
CH2Cl2:MeOH										
95:5	118-120:MLM15	100	100	100	50	25	25	25	0	62.5
	121-128:MLM16	75	50	25	0	0	0	0	0	250
90:10	129-150:MLM17	100	50	0	0	0	0	0	0	250
80:20										
70:30										
60:40										

MLM: *M. lucida* leaves. The 0 μg/ml are the negative control wells with 2% DMSO.

### Cytotoxicity and acute toxicity of active extracts

**Figure 7:**
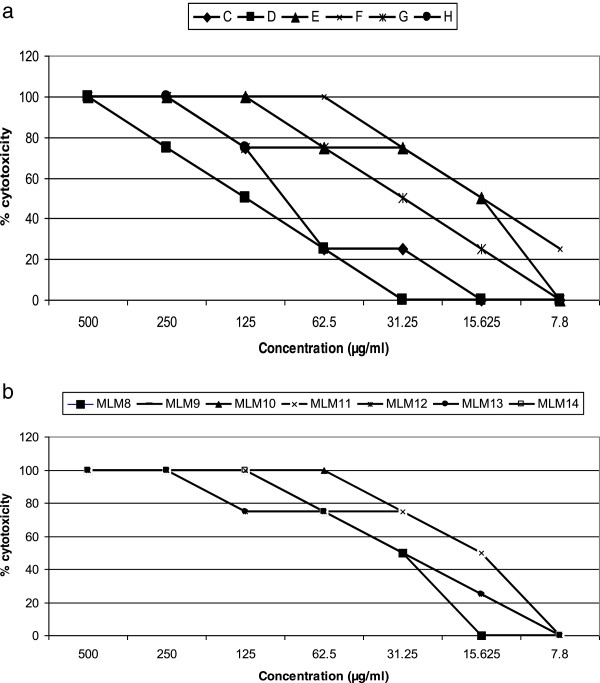
**% cytotoxicity of most active fractions of** ***C. laurinum*** **(a) and** ***M. lucida*** **(b) on LLCMK2 cells.**

Cytotoxicity was determined for all the crude extracts and chromatographic fractions*,* while acute toxicity was done only for the most active extracts and fractions. For the most active fractions of *C. laurinum*, the cytotoxic concentration of the fractions on LLCMK2 cells ranged from 15.125 μg/ml to 125 μg/ml (Figure 7a) while those of fractions from *M. lucida* ranged from 15.625 μg/ml to 31.25 μg/ml (Figure 7b). The CC_50s_ of the active fractions for *C. laurinum* and *M. lucida* are respectively shown on Figures 3 and 5. The SIs of the two active extracts were greater than that of ivermectin. All the animals for acute toxicity were monitored daily for changes in physical activities and appearance. Immediately following administration of the test substances, the animals displayed signs of discomfort and feebleness which lasted only for few minutes. By the first observation (30 minutes after dosing), the animals were already agile. For the fourteen days study period, none of the dosed mice died. When compared with their normal controlled counterparts, the intake of food and water was the same. There was daily regular agility. No change was observed in the physical activities and behavior in the test animal. Additionally, there was no loss of fur, no change in skin and mucous membrane. Visibly, the test and controlled groups were indistinguishable from one another on basis of their appearances and physical activity at the end of the 14 days study period.

### Phytochemical constituents of most active fractions

Only fraction MLM14 from *M. lucida* showed a positive test for alkaloids. On the other hand, sterols were present in all the active fractions of *C. laurinum* as well as fractions MLM13 and MLM14 of *M. lucida* (Table 4).

**Table 4:** **Phytochemical analysis of the most active fractions**

	Most active fractions from ***C. laurinum***						Most active fractions from ***M. lucida***				
Active substance	C	D	E	F	G	H	MLM9	MLM10	MLM11	MLM13	MLM14
**Alkaloids**	-	-	-	-	-	-	-	-	-	-	+
**Sterols**	+	+	+	+	+	+	+	-	-	+	+
**Triterpenes**	-	-	-	-	-	-	-	+	+	-	-
**Saponins**	-	-	-	-	-	-	-	-	-	-	+
**Tannins**	-	-	-	-	-	-	-	-	-	-	-
**Flavonoids**	-	-	-	-	-	-	+	-	-	+	+

## Discussion

The primary aim of this study was to search in medicinal plants for new filaricidal compounds that might serve as drug leads. From ethnobotanical survey, two plants, *C. laurinum* and *M. lucida* from the same family (*Rubiaceae*) were selected for study. Traditional health practitioners in Finge, a small community in North West Cameroon with high prevalence of onchocerciasis use *C. laurinum* to manage the ailment. This plant showed activity when screened in vitro against the bovine form of the parasite, *O. ochengi* which is the closest relative to the human form, *O. volvulus*. Based on the results obtained with *C. laurinum*, we decided to extend the study using a different member of the family, *M. lucida* gotten from the foot of Mount Cameroon (a different ecological setting). The results obtained from this study revealed the presence of filaricidal activities in the two plants that were obtained from two different geographical regions. In separate studies, both plants have been reported to show activites against *Trypanosoma brucei brucei*, a range of bacterial species, *Leishmania major* and *P. falciparum* [24–26, 36]. Other studies carried out with *Carapa procera, Polyalthia suaveolens* [21], *Piliostigma thonningii, Ocimum gratissimum, Nauclea latifolia* and *Alstonia boonei* [37], *Homalium Africanum* [22], *Annona senegalensis, Anogeissus leiocarpus*, *Euphorbia hirta*, *Parquetina nigrescens* and *Khaya senegalensis* [23] have demonstrated anti-*Onchocerca* and anthelmintic activities.

The methanolic extracts of the leaves were the most active of both plants. Traditionally, *C. laurinum* concoction is prepared by boiling the leaves of the plant in water. Thereafter, a glass (~200 ml) of the resulting liquid is drunk three times a day for upto one month depending on manifested signs and symptoms. The highest activity recorded by the methanolic extracts partly justifies the efficiency of this method of preparation of the traditional regimen although the traditional preparations are hardly standardized. In a different study [22], the non-polar extracts of *Homalium africanum* were shown to be more active than the polar extracts. Although studies have shown that non-polar compounds such as essential oils are nematocidal [38], we herein demonstrate the nematocidal property of some polar extracts. Ndjonka and others reported similar findings in which the ethanolic extract from the leaves of *Annona senegalensis* killed *O. ochengi* worms [23]. Significant differences were observed in the IC_50s_ of the crude extract and their fractions. The IC_50s_ of *C. laurinum* crude extract on mfs were 93.75 μg/ml and 15.625 μg/ml respectively, for the crude extract and fraction G (fraction with highest activity). The same observation was seen with *M. lucida* extract which had IC_50s_ of 62.5 μg/ml and 7.8 μg/ml for its crude extract and most active fraction (MLM 10) respectively. In like manner, IC_50s_ of adult worms varied considerably with the derived fractions and the stock crude extracts. For both plants, the IC_50s_ of the most active fractions were at least three fold lower than that of the extract. At present, we are investigating and characterizing the bioactive compounds responsible for the observed filaricidal activity. It is likely that following purification, some compounds will be obtained that will demonstrate activity that is higher than those gotten from their fractions.

Activity varied between the stages of the parasite and the extract/fraction used. While some extracts/fractions exerted more activity on the male adult worms, some showed more activity on the female and others still demonstrated more efficiency only on the mfs. The fractions also demonstrated a dose- and time-dependent activity with efficacy increasing when the dose was increased and the time of incubation also extended. Discrepancy in activity between the various stages of the parasite probably indicates a variation in the target of the different extracts. However, all the extracts/fractions that were either active or moderately active on adult worm also showed activity on microfilariae. The reverse was not always true. This juvenile form of the parasite is apparently more susceptible to the extracts than their adult worm counterpart. Ivermectin exerts a similar selective effect. One fraction from *C. laurinum* (fraction E) had the same IC_50_ (62.5 μg/ml) for all the three forms of the parasite. This was in contrast to the rest of the fractions where IC_50s_ for mfs were generally lower than IC_50s_ of adult worms. These interesting findings give more optimisms in the *Onchocerca* drug search as the drug of choice for the treatment of onchocerciasis should be one that possesses both macro-/microfilaricidal activity.

The studied plants showed no overt toxicity in laboratory mice. Regular food and water intake by all the mice was not hampered during the fourteen day period of follow-up. The apparent weakness and discomfort noticed immediately after dosing was probably a result of shock that came about by the consecutive use of 70% alcohol (for disinfecting the site of administration) and injection. Contrary to this observation, the most active extracts/fractions were apparently cytotoxic to LLCMK2 cells in the in vitro assay. The SI values for both the methanolic extracts of *M. lucida* and *C. laurinum* were 1.52 μg/ml and 2.67 μg/ml respectively while that of the control drug; ivermectin was 1 μg/ml. This finding suggests that cytotoxicity of an extract/compound might not necessarily imply in vivo toxicity. This finding is consistent with the observations on *Homalium africanum* and *Margaritaria discoidea* [22]. A detoxification mechanism may account for this in vivo observation and also partly justifies the absence of adverse effects in patients who take the concoction for therapy.

## Conclusion

This study has revealed the anti-*Onchocerca* activities of extracts of *M. lucida* and *C. laurinum*, indicating a possible new source for developing a phytomedicine or drug for the treatment of the disease. It also validates the use of *C. laurinum* by traditional health practitioners in the management of the disease in rural areas where onchocerciasis is prevalent.
